# Real-World Safety of Niraparib for Maintenance Treatment of Ovarian Cancer in Canada

**DOI:** 10.3390/curroncol31060264

**Published:** 2024-06-19

**Authors:** Qi Guan, Suriya J. Aktar, Reka E. Pataky, Mariet Mathew Stephen, Maud Marques, Karen Gambaro, Kahina Rachedi, Katharina Forster, Samara Strub, David Stock, Louis de Léséleuc, Winson Y. Cheung, Stuart Peacock, Christie Farrer, Scott Gavura, Mina Tadrous, Robert C. Grant, Kelvin K. W. Chan

**Affiliations:** 1 Ontario Health, Toronto, ON M5G 2L3, Canada; 2 Canadian Cancer Real-World Evaluation Platform, Toronto, ON M5G 2L3, Canada; 3 Canadian Centre for Applied Research in Cancer Control, Toronto, ON M5G 2L3, Canada; 4 BC Cancer, Vancouver, BC V5Z 4E6, Canada; 5 Oncology Outcomes, Calgary, AB T2N 1N4, Canada; 6 Alberta Health Services, Edmonton, AB T5J 3E4, Canada; 7 Department of Oncology, Tom Baker Cancer Centre, University of Calgary, Calgary, AB T2N 1N4, Canada; 8 Exactis Innovation, Montreal, QC H3T 1Y6, Canada; 9 CADTH, Ottawa, ON K1S 5S8, Canada; 10 Faculty of Health Sciences, Simon Fraser University, Burnaby, BC V5A 1S6, Canada; 11 Leslie Dan Faculty of Pharmacy, University of Toronto, Toronto, ON M5S 1A1, Canada; 12 Women’s College Hospital, Toronto, ON M5S 1B2, Canada; 13 ICES, Toronto, ON M4N 3M5, Canada; 14 Princess Margaret Cancer Centre, Toronto, ON M5G 2C4, Canada; 15 Department of Medicine, University of Toronto, Toronto, ON M5S 1A1, Canada; 16 Sunnybrook Health Sciences Centre, Toronto, ON M4N 3M5, Canada

**Keywords:** ovarian cancer, niraparib, real-world evidence

## Abstract

Niraparib was recently funded in Canada for the maintenance treatment of ovarian cancer following platinum-based chemotherapy. However, the drug’s safety profile in the real world remains uncertain. We conducted a cohort study to describe the patient population using niraparib and the proportion that experienced adverse events between June 2019 and December 2022 in four Canadian provinces (Ontario, Alberta, British Columbia [BC], and Quebec). We used administrative data and electronic medical records from Ontario Health, Alberta Health Services, and BC Cancer, and registry data from Exactis Innovation. We summarized baseline characteristics using descriptive statistics and reported safety outcomes using cumulative incidence. We identified 514 patients receiving niraparib. Mean age was 67 years and most were initiated on a daily dose of 100 or 200 mg/day. Grade 3/4 anemia, neutropenia, and thrombocytopenia occurred in 11–16% of the cohort. In Ontario, the three-month cumulative incidence of grade 3/4 thrombocytopenia was 11.6% (95% CI, 8.3–15.4%), neutropenia was 7.1% (95% CI, 4.6–10.4%), and anemia was 11.3% (95% CI, 8.0–15.2%). Cumulative incidences in the remaining provinces were similar. Initial daily dose and proportions of hematological adverse events were low in the real world and may be related to cautious prescribing and close monitoring by clinicians.

## 1. Introduction

Ovarian cancer is the tenth most commonly diagnosed cancer amongst women in Canada; however, it ranks fifth in cancer mortality [1]. Because patients often present with non-specific symptoms and experience rapid disease spread throughout the abdomen, many individuals are diagnosed at an advanced stage and have a poor prognosis [2]. The 5-year survival rate for ovarian cancer in Canadian women is approximately 45% [3], and major risk factors include genetic mutations, familial history, obesity, smoking, endometriosis, and older age [4].

Maintenance treatment using poly (adenosine diphosphate [ADP]-ribose) polymerase (PARP) inhibitors after response to platinum-based chemotherapy has shown improvement in progression-free survival for patients with new and recurrent ovarian cancer, as compared to placebo [2,5]. Although evidence of the efficacy of PARP inhibitors is strongest amongst patients with mutations in BReast CAncer (*BRCA*) genes 1 and 2, data from clinical trials [6,7,8] and real-world evidence [9,10] have shown potential benefit for patients without such mutations as well. As such, Health Canada approved the use of niraparib, an oral PARP inhibitor, for the maintenance treatment of patients with recurrent ovarian cancer in 2019 [11] and newly diagnosed ovarian cancer in 2020 [12], regardless of a patient’s *BRCA* mutation status. Niraparib was subsequently added to the public drug formularies of provinces across Canada [13,14,15,16].

Although clinical trials showed manageable levels of adverse events associated with the use of niraparib [6,7,8], the proportion of such outcomes occurring in real-world settings in Canada is currently unknown. Participant eligibility is highly strict in clinical trials and therefore patient populations and adverse event rates may differ between clinical trials and real-world settings [17,18]. Thus, Ontario Health submitted a query to the newly-created CADTH CoLab Network [19] to understand the use of niraparib in the real world. We (the Canadian Cancer Real-world Evaluation [CCRE] Platform [20] and Exactis Innovation [21]) are part of the CoLab Network and were commissioned to characterize the patient population using niraparib for maintenance treatment of ovarian cancer in four Canadian provinces and to determine the proportion of these patients who experience adverse events. 

## 2. Materials and Methods

### 2.1. Population and Setting

We identified a cohort of patients who were ≥18 years of age and undergoing maintenance treatment of newly diagnosed or recurrent ovarian cancer after response to platinum-based chemotherapy using publicly funded niraparib in Ontario, Alberta, and British Columbia (BC), as well as all adult patients in Quebec who were enrolled in the Personalize My Treatment registry and using niraparib for the above indication. The study period ranged from 27 June 2019 to 31 December 2022; however, the accrual window varied by province due to differences in data availability and implementation dates for public funding of niraparib. The accrual window began on 27 June 2019 in Ontario, 1 January 2020 in Quebec, 1 December 2021 in BC, and 1 January 2022 in Alberta. We excluded patients if they had invalid critical information (i.e., patient identification number, death date, province of residence, treatment date), and in BC, we excluded individuals who were treated outside BC Cancer regional cancer centres as the hematological outcomes for these patients were not available. We also excluded patients in BC who initiated niraparib privately before the drug was publicly funded.

### 2.2. Study Design

We conducted a historical, single-arm, cohort study to determine the safety of niraparib for the maintenance treatment of newly diagnosed or recurrent ovarian cancer amongst patients in Ontario, Alberta, BC, and Quebec who respond to platinum-based chemotherapy. Response to platinum-based chemotherapy is a criterion for public funding of niraparib in many Canadian jurisdictions, although there may be a small group of case-by-case assessments that may result in funding for patients who cannot tolerate platinum-based chemotherapy. In this study, it is assumed that all patients who received publicly funded niraparib have met the platinum-based chemotherapy criterion or have received an exemption. We identified the first date of niraparib dispensing for each patient (index date) and followed the cohort until the first of treatment discontinuation, defined as last date of niraparib use plus 60 days, death, or study period end (31 December 2022) to look for safety outcomes. We used a lookback window of up to 5 years prior to the index date to ascertain previously diagnosed comorbidities. 

### 2.3. Data Sources

We used linked administrative health databases, electronic medical records, and registry data for this study. The CCRE Platform’s access to Ontario data is governed under section 45 of the province’s Personal Health Information Protection Act and is not subject to additional review by an ethics review board. Linked administrative databases for Ontario are housed at Ontario Health and contain data on instances of interactions with the healthcare system including but not limited to the dispensing of publicly funded prescription medications, diagnoses and procedures in the inpatient and outpatient setting, and records of laboratory tests. A detailed description of the databases used is provided in Table S1 of the Supplementary Materials. Research activities at the Alberta site of the CCRE Platform were approved by the Health Research Ethics Board of Alberta—Cancer Committee and data access was approved by Alberta Health Services with a data disclosure agreement. Data sources included the Pharmacy Information Network (PIN), electronic medical records (EMRs), and additional variables collected through chart abstraction. Research activities at the BC site of the CCRE Platform were approved by the University of British Columbia—BC Cancer Research Ethics Board and data access was approved by the BC Cancer Data Stewards. Data sources in BC included the BC Cancer Registry, the Provincial Systemic Therapy Program, and additional variables (treatment history and laboratory results) collected through chart review. Data for Quebec patients were ascertained from the Personalize My Treatment registry, which is an active cancer registry developed and managed by Exactis Innovation that collects molecular and clinical data for cancer patients at 16 sites across Canada [22]. Ethics approval for this registry was provided by the CIUSSS West-Central Montreal Research Ethics Board (REB number: MP-05-2016-321). Due to provincial privacy policies to protect patient confidentiality, administrative data in Ontario, Alberta, and BC are subject to censoring when counts are below 6 (Ontario and BC) or 10 (Alberta). Thus, small cell numbers in these jurisdictions will be masked with <6 or <10 where necessary. Additional masking of cells may be completed to avoid back-calculation of small cells. Although the Personalize My Treatment Registry is not subject to the same privacy regulations, we masked values <6 to maintain consistency.

### 2.4. Key Study Measures

#### 2.4.1. Exposure

The exposure of interest in this study is the use of niraparib for maintenance treatment of ovarian cancer. This is ascertained by identifying a dispensing of the oral medication (DIN: 02489783, the only niraparib product marketed in Canada as per Health Canada at the time of our study) in publicly funded prescription drug reimbursement records in Ontario, electronic medical records and pharmacy dispensing records in BC and Alberta, and patient charts in Quebec. Three additional DINs were approved by Health Canada in the fall of 2023; however, these medications were not included in the current analysis as their introduction to the Canadian market was outside of the study period.

#### 2.4.2. Outcomes

We reported a set of hematological adverse events as the primary outcomes between index date and 31 December 2022. These included thrombocytopenia, neutropenia, and anemia, which were defined based on platelet, neutrophil, and hemoglobin levels (respectively) according to the Common Terminology Criteria for Adverse Events, version 5 [23]. We also reported a set of secondary outcomes during the same time period, including febrile neutropenia, incident hypertension, blood transfusion (any, platelet, and red blood cell), hospitalizations, emergency department visits, treatment discontinuation, and median follow-up time. See Table S2 in the Supplementary Materials for additional details on variable definitions.

### 2.5. Statistical Analyses

We characterized the patient population in each province using descriptive statistics and constructed cumulative incidence function curves for primary outcomes. When calculating cumulative incidence, we accounted for treatment discontinuation and death as competing risks and censored on the end of study period, using the Fine–Gray model [24]. Where possible, we combined aggregate values from each province in order to report on an “all provinces” measure for baseline characteristics and secondary outcomes. We conducted all analyses in BC and Ontario using SAS 9.4 (SAS Institute, Cary, NC, USA) and all analyses in Alberta and Quebec using R (v4.2.2 in Alberta and v4.3.0 in Quebec; R Foundation for Statistical Computing, Vienna, Austria). 

#### Supplementary Analysis

Following the main analyses, we conducted additional analysis in Ontario to determine the mean number of blood tests conducted for patients during the first 35 days of treatment as a measure of clinical monitoring, explored potential changes in dose throughout treatment for patients in the cohort, and stratified the hematological outcomes by initial daily dose of niraparib. This analysis was restricted to the Ontario cohort due to data availability and sample size.

## 3. Results

### 3.1. Cohort Characteristics

**Figure 1:**
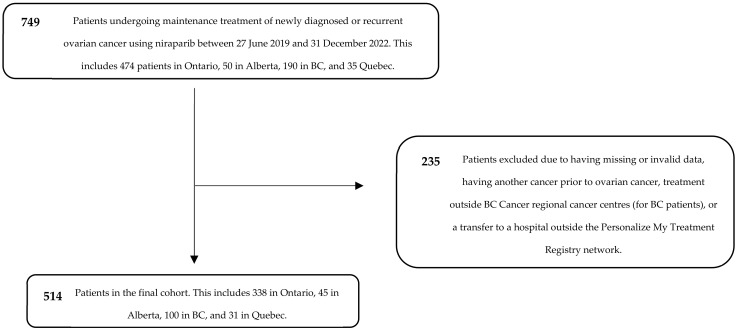
CONSORT diagram for cohort creation.

We identified 749 patients who received niraparib for maintenance treatment of newly diagnosed or recurrent ovarian cancer between 2019 and 2022, following response to platinum-based chemotherapy, in Ontario, Alberta, BC, and Quebec. Following the exclusion of patients due to data quality and issues in treatment location, 514 patients remained in the final cohort. This included 338 patients in Ontario, 45 in Alberta, 100 in BC, and 31 in Quebec (Figure 1).

We found that patient characteristics were generally consistent across jurisdictions (Table 1). When examining the cohort as a whole, we observed a mean age of 67 years (±standard deviation of 10 years), and that almost three quarters of patients were originally diagnosed with ovarian cancer between 2020 and 2022 (*N* = 381, 74.1%). Most patients started niraparib maintenance treatment in 2022 (*N* = 459–463, 89.3–90.1%), the ovaries were the most common primary tumour location (*N* = 400–404, 77.8–78.6%), and the majority of tumours presented with serous histology (*N* = 453–461, 88.1–89.7%). On average, patients had 6.5 cycles (±standard deviation of 2.9 cycles) of platinum-based chemotherapy prior to starting niraparib treatment, and over two-thirds of the cohort started on an initial daily dose of 200 mg per day (Table 2).

### 3.2. Primary and Secondary Outcomes

We report crude proportions of hematological adverse events in Table 2. Overall, anemia was the most common primary outcome with 78.0% of the cohort (*N* = 384) experiencing hemoglobin counts lower than normal (i.e., anemia of any grade). Thrombocytopenia and neutropenia of any grade occurred in 42.5% and 39.0% of the overall cohort, respectively. The ranking sequence was similar when examining severe hematological adverse events (i.e., grade 3/4 events); 13.4% (*N* = 66) of the overall cohort experienced severe anemia, 13.2% (*N* = 65) experienced severe thrombocytopenia, and 12.2% (*N* = 60) experienced severe neutropenia. 

**Figure 2:**
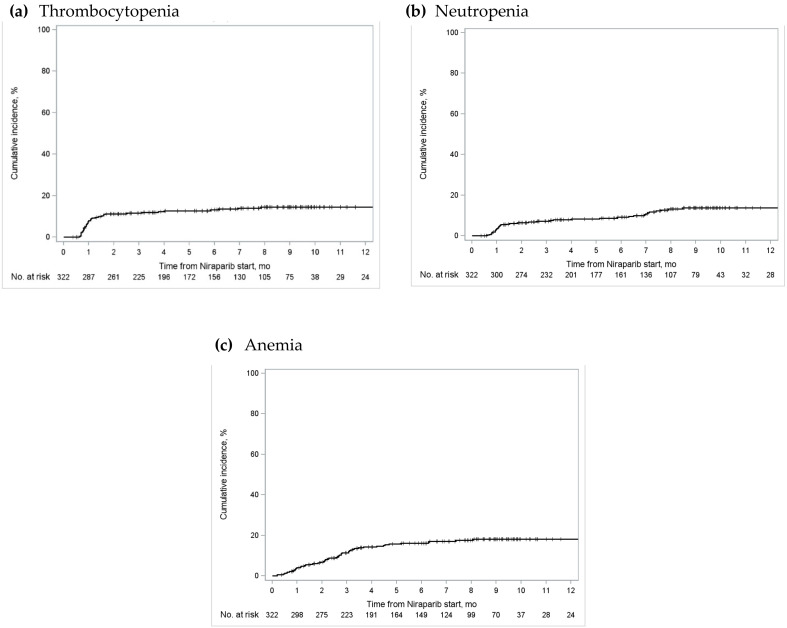
Cumulative incidence of grade 3/4 (**a**) thrombocytopenia; (**b**) neutropenia; and (**c**) anemia in Ontario. The “number at risk” below each figure represents the number of patients who were eligible for the outcome of interest (i.e., patients who received publicly funded niraparib for the maintenance treatment of ovarian cancer, who had not experienced the outcome nor been censored) at various time periods [25].

The 3-month cumulative incidence of severe thrombocytopenia in Ontario was 11.6% (95% confidence interval [CI] 8.3–15.4%), severe neutropenia was 7.1% (95% CI 4.6–10.4%), and severe anemia was 11.3% (95% CI 8.0–15.2%) (Figure 2; Figure S1 and Table S2 in Supplementary Materials). Cumulative incidence of these events plateaued by approximately the eighth month after treatment initiation. Similar trends were observed in Alberta, BC, and Quebec (Figures S2–S4 and Tables S4–S6 in Supplementary Materials).

**Table 2:** Hematological adverse events, stratified by province.

	All Provinces *N* = 426 (%)		Ontario *N* = 322 (%) ^1^		Alberta *N* = 45 (%)		British Columbia *N* = 93 (%) ^1^		Quebec *N* = 31 (%)	
	Any Grade	Grade 3/4	Any Grade	Grade 3/4	Any Grade	Grade 3/4	Any Grade	Grade 3/4	Any Grade	Grade 3/4
Thrombocytopenia	209 (42.5)	65 (13.2)	136 (42.2)	43 (13.4)	16 (35.6)	<10	43 (46.2)	13 (14.0)	14 (45.2)	<6
Neutropenia	192 (39.0)	60 (12.2)	107 (33.2)	37 (11.5)	23 (51.1)	<10	45 (48.4)	13 (14.0)	17 (54.8)	<6
Anemia	384 (78.0)	66 (13.4)	258 (80.1)	52 (16.2)	34 (75.6)	<10	7 (76.3)	8 (8.6)	21 (67.7)	<6

^1^ Laboratory test data available for 322 patients in Ontario and 93 patients in BC.

We reported a number of secondary outcomes that occurred between index date and the study end date for the cohort (Table 3). Very few individuals (<10) experienced febrile neutropenia, and amongst those who did not have a previous diagnosis of hypertension before the index date, approximately 20% (*N* = 44–52, 19.4–22.9%) were newly diagnosed with hypertension. Over one-tenth of the overall cohort received a blood transfusion during the observation window (*N* = 53, 12.8%); however, the proportion in Ontario was lower (*N* = 33, 9.8%) than that in Alberta (*N* = 11, 24.4%) and Quebec (*N* = 9, 29.0%) when this variable was reported by province. Approximately one-fifth of the overall cohort was hospitalized for any reason (*N* = 80, 19.3%) and over one-third of the group had an emergency department visit (*N* = 153–157, 37.0–37.9%). Over one-third of the cohort discontinued niraparib maintenance treatment, with a mean time to discontinuation of 164 days (±standard deviation of 111 days).

### 3.3. Supplementary Analyses

In our additional exploratory analysis of the Ontario cohort, we found that patients received a mean of 3 blood tests in the first 35 days of niraparib maintenance treatment (2.9 ± standard deviation of 2.2) and this varied slightly when stratified by initial daily dose of niraparib. Patients who started treatment at 100 mg per day received a mean of 2.7 blood tests during the first 35 days of treatment (±standard deviation of 3.0 tests), while those who started at 200 mg per day received a mean of 2.9 blood tests (±standard deviation of 1.7 blood tests) and those who started at 300 mg per day received a mean of 3.8 blood tests (±standard deviation of 2.1) during the same period. Among the patients who started at a dose of 100 mg per day (*N* = 58), we found that the majority remained on a dose of 100 mg per day throughout their entire treatment (*N* = 51, 87.9%). This number decreased amongst those who started on 200 mg/day, with 67% staying on 200 mg/day (*N* = 118). For those who started on 300 mg/day, only 40% (*N* = 8) remained on the same dose throughout their treatment while 60% (*N* = 12) required a dose decrease. When we stratified the cumulative incidence curves by initial niraparib dose, we found that a lower initial dose appeared to be related to lower hematological toxicities numerically but this did not reach statistical significance (*p*-value = 0.09 for thrombocytopenia, 0.05 for neutropenia, and 0.11 for anemia; Supplementary Materials, Figure S5 and Table S7).

## 4. Discussion

### 4.1. Summary

In this historical cohort study of patients on niraparib for the maintenance treatment of ovarian cancer in four Canadian provinces, we identified 514 patients receiving treatment between June 2019 and December 2022 (338 in Ontario, 45 in Alberta, 100 in BC, and 31 in Quebec) [26]. Initial daily dose of niraparib varied amongst the cohort, with approximately 70% starting on 200 mg per day, over 20% starting on 100 mg per day, and just under 10% starting on 300 mg per day. Although most of the cohort experienced a hematological adverse event of any grade, severe events (grade 3/4 level) occurred in a small portion of the group.

### 4.2. Comparison with Existing Literature

Overall, we found that the proportion of severe hematological adverse events in the Canadian real-world settings was lower than in the two seminal clinical trials on which Canadian regulatory bodies based their approval for niraparib [6,7]. Given that baseline patient characteristics of the trial cohorts were generally similar to those of the current study (although our cohort was slightly older), it is possible that these differences may be attributable to differences in initial dosing strategies. In the NOVA trial examining the use of niraparib amongst patients with recurrent ovarian cancer, all participants were initiated on the standard 300 mg per day dose and the proportion of severe hematological adverse events were substantial (33.8% experienced grade 3/4 thrombocytopenia, 19.6% experienced grade 3/4 neutropenia, and 25.3% experienced grade 3/4 anemia). These findings raised initial awareness about the toxicity profile of niraparib [6]. Similarly, the original protocol for the PRIMA study examining niraparib use in patients with newly diagnosed ovarian cancer also implemented a standard 300 mg per day dose, but this was later altered to individualized dosing where patients with a body weight of <77 kg or a platelet count of <150 × 10^9^/L may be initiated on 200 mg per day [7]. This change led to a lower proportion of severe hematological adverse events with the exception of anemia (28.7% experienced grade 3/4 thrombocytopenia, 12.8% experienced grade 3/4 neutropenia, and 31.0% experienced grade 3/4 anemia) [7]. Since the approval of niraparib in Canada, an additional phase III clinical trial was published, examining the efficacy and safety of individualized niraparib dosing (NORA trial) [8]. The cohort characteristics in that trial were similar to those of the current study; however, the average age was approximately 10 years younger. The reported proportions of severe hematological adverse events in that trial were low as well; approximately 11% of the cohort experienced grade 3/4 thrombocytopenia, 20% experienced grade 3/4 neutropenia, and 15% experienced grade 3/4 anemia [8], which were comparable to our study findings.

The few real-world evidence studies published on the safety of niraparib have found that individualized dosing may be helpful in reducing adverse events [9,10,27]. Studies from Norway, China, and Japan have found that thrombocytopenia, neutropenia, and anemia occur within the 10–20% range of the cohort, which is also comparable to our study findings. Although we did not have access to data on patient weight and therefore cannot be certain that individualized dosing was applied consistently across our cohort, approximately one-quarter of our cohort initiated treatment on 100 mg of niraparib per day and most of this group remained on this dose for the duration of treatment. Given that 100 mg per day is not a dose recommended in the product monograph, this may suggest that the low proportions of severe hematological adverse events in our study could be due to clinicians taking a cautious approach to prescribing.

However, patients using niraparib in the real world are not free of adverse events. Clinicians likely have been proactively monitoring for and managing adverse events. In a letter to the editor in early 2023 describing real-world outcomes of niraparib use, Li and colleagues suggested that niraparib can be well tolerated with strict follow-up and careful management of adverse events [28]. This is supported by the results of our ad hoc exploratory analysis showing an average of three blood tests per patient in the first month of treatment. Based on the product monograph, it is suggested that bloodwork should occur weekly during the first month of treatment [29]. Additionally, Li and colleagues also noted that patients who initiate niraparib maintenance treatment more than 20 days after their last chemotherapy treatment were less likely to experience adverse events when compared to those who start in under 20 days. This may be an additional contributing factor to low proportions of severe adverse events in the current study, as the mean number of days between last chemotherapy and niraparib start in our cohort was greater than 20 days.

### 4.3. Implications for Future Research

The unexpected low initial daily doses of niraparib observed in the Canadian real-world settings lead to a number of directions for future research. First, it may be important to determine the clinician and patient factors associated with initiating a low dose of niraparib that is not listed in the product monograph. Furthermore, given that our analysis showed that most patients starting on 100 mg of niraparib per day continued on this dose for the duration of their treatment, it is crucial to determine the effectiveness of initiating and subsequently maintaining a low dose. Should there be substantial impacts on effectiveness when using a 100 mg per day dose, then clinician engagement activities must be developed to promote appropriate prescribing while providing regular blood tests to monitor patients for potential adverse events. Finally, future work should stratify patients based on disease status (i.e., primary vs. recurrent ovarian cancer) as it may be possible that patient outcomes may differ between the two groups.

### 4.4. Limitations

This study had several limitations that warrant further discussion. First, although three-quarters of the cohort (Ontario, Alberta, and BC) were population-based, there remained some province-specific factors that may limit the generalizability of our results. In Ontario, the cohort was derived using patients treated with publicly funded niraparib, which excluded individuals who may have paid for niraparib out-of-pocket or through private insurance. In BC, the cohort did not include patients who were treated outside BC Cancer regional cancer centres, and patients in Quebec were ascertained using a registry which may have been subject to selection bias. However, given that our results remained generally consistent across the four provinces, these issues may have only minimally limited the generalizability of our results. Second, the observation window of our study was limited for some patients due to the recent introduction of niraparib to the public drug formulary. In order to accrue the maximum number of patients into the study, the end of our accrual window coincided with the end of our observation window. This allowed us to report a more fulsome description of the patient population, but it may be possible that we undercounted the crude number of adverse events for patients who initiated niraparib towards the end of the accrual window. However, the use of cumulative incidence for primary outcomes allowed us to overcome time-dependent biases in the estimates. Finally, we did not have access to patient weight for this study and we were therefore unable to determine whether patients starting on 200 mg per day of niraparib were being treated with an individualized dose based on their platelet count and body weight or a dose that differed from clinical recommendations. However, we observed almost one quarter of the cohort initiating on 100 mg per day, which is lower than recommendations from the manufacturer’s product monograph [29] and may thus be considered a low dose.

## 5. Conclusions

In conclusion, the current study examining the use of niraparib for maintenance treatment of newly diagnosed and recurrent ovarian cancer shows that niraparib is used cautiously and at low initial doses in Canadian real-world settings. It is therefore possible that this, in combination with close physician monitoring via frequent blood tests, may be amongst the factors that contributed to low proportions of severe hematological adverse events. Future work should examine the patient and clinician factors associated with initiating on low doses, as well as the effectiveness of initiating and maintaining a low dose of niraparib, in order to guide clinician decision-making throughout the maintenance treatment of ovarian cancer with this drug.

## Figures and Tables

**Table 1:** Baseline characteristics of study cohort, stratified by province.

		All Provinces *N* = 514 (%)	Ontario *N* = 338 (%)	Alberta *N* = 45 (%)	British Columbia *N* = 100 (%)	Quebec *N* = 31 (%)
Mean Age (± SD)		66.8 ± 10.3	68.8 ± 9.7	67.0 ± 9.0	66.1 ± 10.4	65.3 ± 11.9
Urban Residence		396–402 (77.0–78.2)	280 (82.8) ^1^	24 (53.3)	92–98 (92.0–98.0)	N/A
Year of Cancer Diagnosis	≤2018	89 (17.3)	81 (24.0)	<10	<6	<6
	2019	45 (8.8)	26 (7.7)	<10	9 (9.0)	<6
	2020	78 (15.2)	60 (17.8)	<10	<6	10 (32.3)
	2021	217 (42.2)	119 (35.2)	28 (62.2)	60 (60.0)	10 (32.3)
	2022	86 (16.7)	52 (15.4)	<10	25 (25.0)	<6
Cancer Stage at Diagnosis	I–II	37 (7.2)	22 (6.5)	<10	6 (6.0)	<6
	III	225 (43.8)	123 (36.4)	29 (64.4)	50 (50.0)	23 (74.2)
	IV	89–101 (17.3–19.6)	56 (16.6)	<10	27 (27.0)	5–9 (16.1–29.0)
	Unknown	155–163 (30.2–31.7)	137 (40.5)	<10	17 (17.0)	0
Year of Niraparib Start	2020–2021	51–55 (9.9–10.7)	32 (9.5)	0	<6	17 (54.8)
	2022	459–463 (89.3–90.1)	306 (90.5)	45 (100.0)	94–98 (94.0–98.0)	14 (45.2)
Primary Tumour Location	Ovaries	400–404 (77.8–78.6)	312 (92.3)	22 (48.9)	40 (40.0)	26–30 (83.9–96.9)
	Fallopian Tubes	78–86 (15.2–16.7)	12 (3.6)	13–21 (28.9–46.7)	53 (53.0)	0
	Other	32 (6.2)	14 (4.1)	<10	7 (7.0)	<6
Primary Tumour Histology	Serous	453–461 (88.1–89.7)	292 (86.4)	41 (91.1)	94–98 (94.0–98.0)	26–30 (83.8–96.8)
	Endometriod	9–17 (1.8–3.3)	8 (2.4)	<10	0	0
	Other	47 (9.1)	38 (11.2)	<10	<6	<6
Cancer Antigen-125 Level >35 units/mL		38 (21.6)	N/A	12 (26.7)	18 (19.0)	8 (25.8)
Prior Platinum-Based Chemotherapy		505 (98.2)	336 (99.4) ^2^	36–44 (80.0–97.8)	100 (100.0)	26–30 (83.9–96.7)
Mean Number of Prior Cycles of Platinum-Based Chemotherapy (±SD)		6.5 ± 2.9	8.8 ± 4.9	4.0 ± 2.0	6.4 ± 1.0	6.6 ± 1.9
Mean Number of Days Between Last Platinum-Based Chemotherapy and Niraparib Start (±SD) ^2^		57.3 ± 25.9	55.3 ± 31.3	60.4 ± 27.0	55.1 ± 21.8	58.3 ± 22.2
Initial Daily Dose of Niraparib ^3^	100 mg	103 (24.1)	58 (22.9)	17 (37.8)	28 (28.0)	0
	200 mg	288–292 (67.3–68.2)	175 (69.2)	28 (62.2)	60 (60.0)	25–29 (83.3–96.7)
	300 mg	33–37 (7.7–8.6)	20 (7.9)	0	12 (12.0)	<6

^1^ Variable has missing data, therefore value does not add up to 100% for Ontario. ^2^ Calculated for patients whose last dose of platinum-based chemotherapy occurred on or after the start of public funding for niraparib in each jurisdiction. This includes *N* = 287 overall, *N* = 166 in Ontario, *N* = 16 in Alberta, *N* = 76 in BC, and *N* = 15 in Quebec. ^3^ Calculated using cohort of *N* = 253 in Ontario and *N* = 30 in Quebec due to missing data on dose in these two jurisdictions. Abbreviations: N/A, not applicable; SD, standard deviation.

**Table 3:** Secondary outcomes, stratified by province.

		All Provinces *N* = 514 (%)	Ontario *N* = 338 (%)	Alberta *N* = 45 (%)	British Columbia *N* = 100 (%)	Quebec *N* = 31 (%)
Febrile Neutropenia		<10	<6	<10	N/A	0
Incident Hypertension ^1^		44–52 (19.4–22.9)	37 (20.2)	<10	N/A	6 (33.3)
Blood Transfusion	Any Cell Type	53 (12.8)	33 (9.8)	11 (24.4)	N/A	9 (29.0)
	Red Blood Cell	32 (7.7)	22 (6.5)	<10	N/A	<6
	Platelet	18 (4.3)	11 (3.3)	<10	N/A	<6
Hospitalization	Any Type	80 (19.3)	63 (18.6)	17 (37.8)	N/A	0
	Unscheduled	57 (15.4)	57 (16.9)	N/A	N/A	0
Emergency Department Visit		153–157 (37.0–37.9)	134 (39.6)	18 (40.0)	N/A	0
Niraparib Treatment Discontinuation		150–159 (35.0–36.9)	86 (34.0)	<10	41 (41.0)	22 (73.3)
Mean Time to Niraparib Treatment Discontinuation in Days (± SD) ^2^		163.6 ± 111.5	164.6 ± 64.1	135.0 ± 78.0	91.0 ± 53.9	263.8 ±191.3
Median Follow-Up Time in Days (IQR)		N/A ^3^	255 (241–267)	299 (170–274)	250 (78–310)	411 (270–585)

^1^ Denominator for this outcome (i.e., patients without prior hypertension diagnosed) was *N* = 183 in Ontario, *N* = 26 in Alberta, and *N* = 18 in Quebec. ^2^ Calculated using a cohort of *N* = 253 in Ontario and *N* = 30 in Quebec due to missing data on dose in these two jurisdictions. ^3^ Access to patient-level data is only available within each jurisdiction and therefore an aggregate median time to follow-up for all provinces is unavailable. Abbreviations: IQR, interquartile range; N/A, not applicable; SD, standard deviation.

## Data Availability

ON: The analyses, conclusions, opinions, and statements expressed herein are solely those of the authors and do not reflect those of the funding or data sources; no endorsement is intended or should be inferred. Parts of this material are based on data and information provided by Ontario Health (OH), which include data and information provided and/or compiled by the Canadian Institute for Health Information and the Ontario Ministry of Health. The opinions, results, views, and conclusions reported in this paper are those of the authors and do not reflect those of OH or its data sources. No endorsement by OH is intended or should be inferred. BC and AB: Access to data provided by the Data Steward(s) is subject to approval, but can be requested for research projects through the Data Steward(s) or their designated service providers. All inferences, opinions, and conclusions drawn in this publication are those of the authors and do not reflect the opinions or policies of the Data Steward(s). QC: The data presented in this study are available upon request to Exactis Innovation (mmarques@exactis.ca).

## Supplementary Materials

The following supporting information can be downloaded at: https://www.mdpi.com/article/10.3390/curroncol31060264/s1, Table S1: Data sources, by province; Table S2: Diagnosis codes for select covariates use in study, stratified by province; Figure S1: Cumulative incidence of grade 3/4 (a) thrombocytopenia; (b) neutropenia; and (c) anemia in Ontario; Table S3: Cumulative incidence at various timepoints for grade 3/4 hematological adverse events in Ontario; Figure S2: Cumulative incidence of grade 3/4 (a) thrombocytopenia; (b) neutropenia; and (c) anemia in Alberta; Table S4: Cumulative incidence at various timepoints for grade 3/4 hematological adverse events in Alberta; Figure S3: Cumulative incidence of grade 3/4 (a) thrombocytopenia; (b) neutropenia; and (c) anemia in BC; Table S5: Cumulative incidence at various timepoints for grade 3/4 hematological adverse events in BC; Figure S4: Cumulative incidence of grade 3/4 (a) thrombocytopenia; (b) neutropenia; and (c) anemia in Quebec; Table S6: Cumulative incidence at various timepoints for grade 3/4 hematological adverse events in Quebec; Figure S5: Cumulative incidence of grade 3/4 (a) thrombocytopenia; (b) neutropenia; and (c) anemia in Ontario, stratified by initial daily dose of niraparib. Note, the total number of patients included is 314, due to missing initial daily dose data and laboratory test data; Table S7: Cumulative incidence at various timepoints for grade 3/4 hematological adverse events in Ontario, stratified by initial daily dose of niraparib.
