# Supplementary material for: Real-World Safety of Niraparib for Maintenance Treatment of Ovarian Cancer in Canada
Source: Curr Oncol. 2024 Jun 19;31(6):3591–602. doi: 10.3390/curroncol31060264 (PMC11202616; doi:10.3390/curroncol31060264)
Supplement: Supplementary file 1 [file curroncol-31-00264-s001.zip › curroncol-3024290-supplementary.pdf]

Supplemental Materials

Table S1. Data sources, by province

Province	Data Sources and Description
Ontario	**Activity Level Reporting**<br>- Records of visits to oncology centres in Ontario<br>- Used for cohort creation and identification of baseline characteristics<br>**Canadian Institute for Health Information (CIHI)-Discharge Abstract Database**<br>- All records of procedures and diagnoses in an inpatient setting<br>- Used for identification of baseline characteristics and outcomes<br>**CIHI-National Ambulatory Care Reporting System Database**<br>- All records of procedures and diagnoses that occur in the ambulatory setting<br>- Used for identification of outcomes<br>**CIHI-Same Day Surgery**<br>- All records of same day surgeries<br>- Used for identification of baseline characteristics and outcomes<br>**New Drug Funding Program**<br>- All records of new and expensive injectable cancer drugs administered in hospitals<br>- Used for identification of baseline characteristics and outcomes<br>**Ontario Cancer Registry**<br>- Records of cancer diagnoses<br>- Used for cohort creation and identification of baseline characteristics<br>**Ontario Drug Benefits Database**<br>- All records of publicly funded prescription medications<br>- Used for cohort creation, identification of baseline characteristics and outcomes<br>**Ontario Health Insurance Plan**<br>- All records of procedures and diagnoses in an outpatient setting<br>- Used for identification of baseline characteristics and outcomes<br>**Ontario Laboratory Information System Database**<br>- All lab records from hospital, community, and public health labs in Ontario<br>- Used for identification of outcomes<br>**Registered Persons Database**<br>- Demographics data<br>- Used for cohort creation, identification of baseline characteristics and outcomes
Alberta	**Electronic Medical Records**<br>- Used for cohort creation, identification of baseline characteristics and outcomes<br>**Pharmaceutical Information Network Database**<br>- All records of prescription medications dispensed in Alberta for all payers<br>- Used for cohort creation
BC	**BC Provincial Systemic Therapy Program**<br>- Pharmacy dispensing records for all publicly-funded systemic therapies<br>- Used for cohort creation, identification of baseline characteristics and outcomes<br>**BC Cancer Registry**<br>- Records of patient demographics, cancer diagnoses and mortality<br>- Used for cohort creation, identification of baseline characteristics and outcomes<br>**Electronic Medical Records**<br>- Used for identification of baseline characteristics and outcomes
Quebec	**Electronic Medical Records**<br>- Used to identify hematological adverse events<br>**Personalize My Treatment Registry**<br>- All electronic medical records in patient charts for participants of the registry

Table S2. Diagnosis codes for select covariates used in study, stratified by province.

Variable	Ontario	Alberta	BC	Quebec
Thrombocytopenia[1]	**Grade 1:** platelet count between 75 and150 x 10^9/L<br>**Grade 2:** platelet count between 50 and <75 x 10^9/L<br>**Grade 3:** platelet count between 25 and <50 x 10^9/L<br>**Grade 4:** platelet count <25 x 10^9/L			
Neutropenia[1]	**Grade 1:** neutrophil count between 1.5 and 2.0 x 109/L<br>**Grade 2**: neutrophil count between 1.0 and <1.5 x 109/L<br>**Grade 3**: neutrophil count between 0.5 and <1.0 x 109/L<br>**Grade 4:** neutrophil count <0.5 x 109/L			
Anemia[1]	**Grade 1:** hemoglobin count between 100 and 120 g/L<br>**Grade 2:** hemoglobin count between 80 and <100 g/L<br>**Grade 3:** hemoglobin count between 65 and <80 g/L<br>**Grade 4:** hemoglobin count <65 g/L			
Febrile neutropenia	Presence of the ICD-10 codes: D70 (most responsible diagnosis) **AND** R50.8 or R50.9 (any diagnosis) **during observation window**	Ascertained using EMR data during the observation window	N/A	Ascertained using EMR data during the observation window
Hypertension	1 hospital admission for hypertension (I10.x, I11.x, I12.x, I13.x, or I15.x in CIHI-DAD) **OR**<br><br>2 physician claims for hypertension (401-405 in OHIP for Ontario, EMR in Alberta) **within *2 years* for prior diagnosis of hypertension.**<br><br>**--**<br><br>1 hospital admission for hypertension (I10.x, I11.x, I12.x, I13.x, or I15.x in CIHI-DAD) **OR**<br><br>2 physician claims for hypertension (401-405 in OHIP for Ontario, EMR in Alberta) ***during the observation window* for incident hypertension.**		N/A	Ascertained using EMR data during the observation window
Time to niraparib discontinuation	Patients are identified as having discontinued treatment if there are more than 60 days between the date of their last treatment (date of last prescription dispensing plus the days' supply of the prescription) and the study end date. This definition only applied to patients who started niraparib more than 60 days before the study end date.	Ascertained using EMR data during the observation window	Same as Ontario	Ascertained using EMR data during the observation window

[1] From Common Terminology Criteria for Adverse Events version 5

(a) Thrombocytopenia

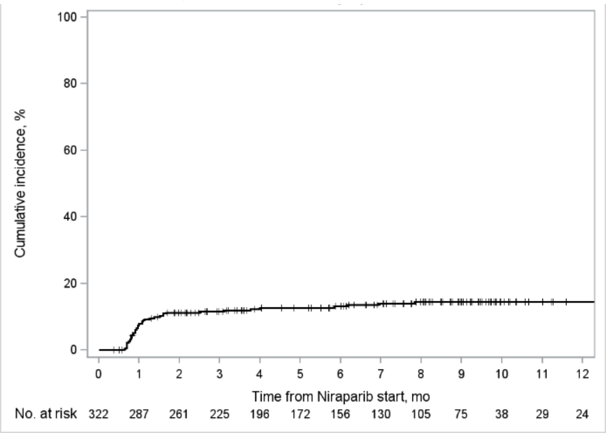

(b) Neutropenia

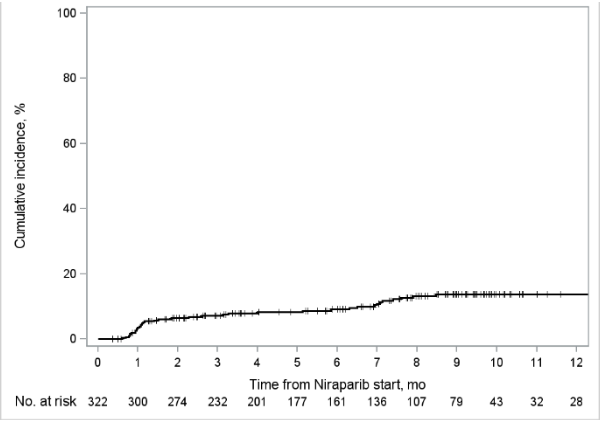

(c) Anemia

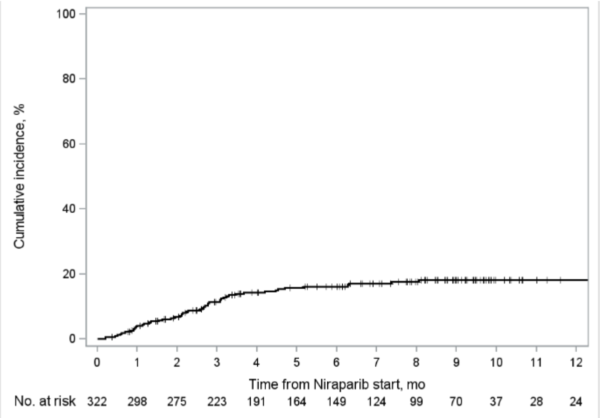

Figure S1. Cumulative incidence of grade 3/4 (**a**) thrombocytopenia; (**b**) neutropenia; and (**c**) anemia in Ontario.

Table S3. Cumulative incidence at various timepoints for grade 3/4 hematological adverse events in Ontario

Outcome	1 Month	2 Months	3 Months	6 Months	9 Months	12 Months
Thrombocytopenia	8.0% (5.3%-11.3%)	11.2% (8.0%-15.0%)	11.6% (8.3%-15.4%)	13.1% (9.6%-17.2%)	14.5% (10.7%-18.8%)	14.5% (10.7%-18.8%)
Neutropenia	3.5% (1.9%-6.0%)	6.4% (4.1%-9.6%)	7.1% (4.6%-10.4%)	9.1% (6.1%-12.7%)	13.7% (9.9%-18.3%)	13.7% (9.9%-18.3%)
Anemia	4.1% (2.3%-6.7%)	6.8% (4.3%-9.9%)	11.3% (8.0%-15.2%)	16.2% (12.2%-20.7%)	18.0% (13.7%-22.8%)	18.0% (13.7%-22.8%)

(a) Thrombocytopenia

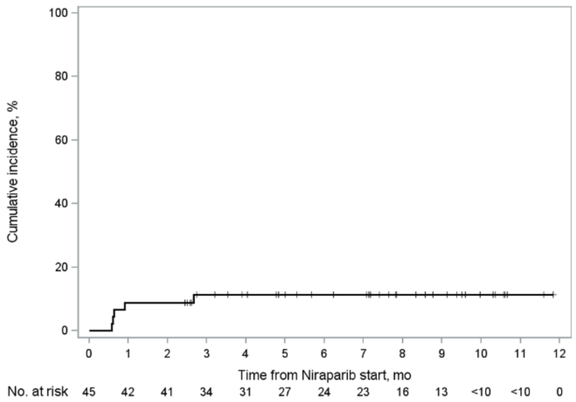

(b) Neutropenia

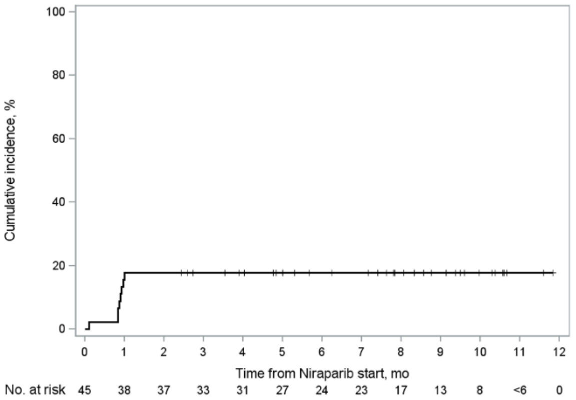

(c) Anemia

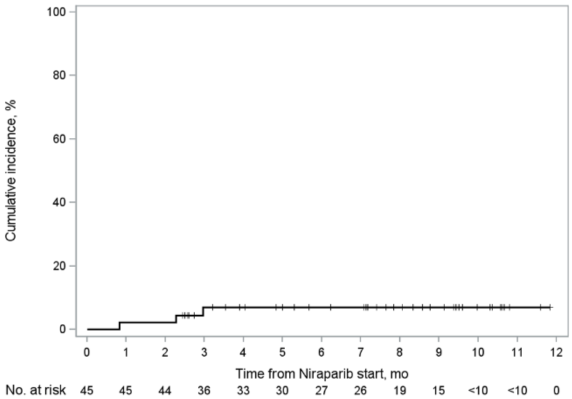

Figure S2. Cumulative incidence of grade 3/4 (**a**) thrombocytopenia; (**b**) neutropenia; and (**c**) anemia in Alberta.

Table S4. Cumulative incidence at various timepoints for grade 3/4 hematological adverse events in Alberta

Outcome	1 Month	2 Months	3 Months	6 Months	9 Months	12 Months
Thrombocytopenia	8.9% (2.8%-19.5%)	8.9% (2.8%-19.5%)	11.4% (4.1%-22.7%)	11.4% (4.1%-22.7%)	11.4% (4.1%-22.7%)	N/A
Neutropenia	17.8% (8.2%-30.3%)	17.8% (8.2%-30.3%)	17.8% (8.2%-30.3%)	17.8% (8.2%-30.3%)	17.8% (8.2%-30.3%)	N/A
Anemia	2.2% (0.2%-10.3%)	2.2% (0.2%-10.3%)	7.0% (1.8%-17.4%)	7.0% (1.8%-17.4%)	7.0% (1.8%-17.4%)	N/A

(a) Thrombocytopenia

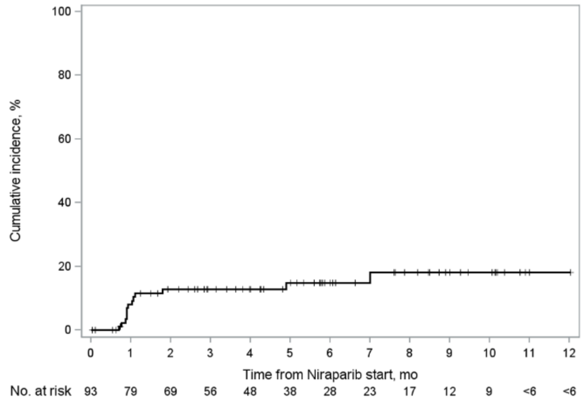

(b) Neutropenia

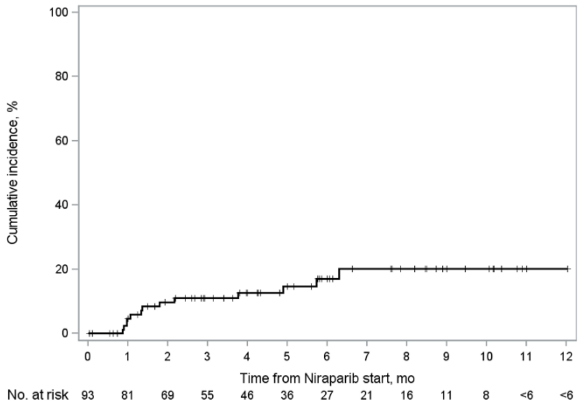

(c) Anemia

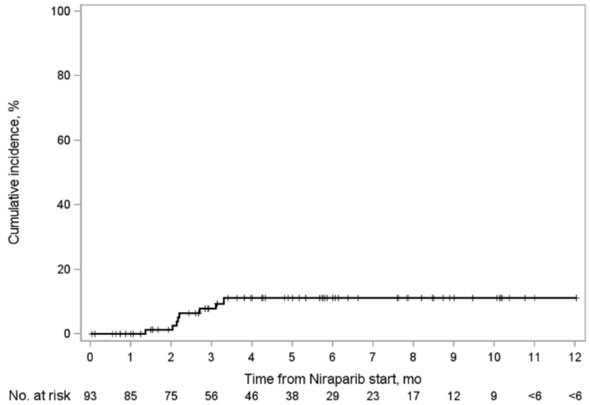

Figure S3. Cumulative incidence of grade 3/4 (**a**) thrombocytopenia; (**b**) neutropenia; and (**c**) anemia in BC.

Table S5. Cumulative incidence at various timepoints for grade 3/4 hematological adverse events in BC

Outcome	1 Month	2 Months	3 Months	6 Months	9 Months	12 Months
Thrombocytopenia	8.1% (3.5% -15.1%)	12.8% (6.8% -20.9%)	12.8% (6.8% -20.9%)	14.8% (8.0% -23.7%)	18.0% (9.4% -28.9%)	18.0% (9.4%-28.9%)
Neutropenia	4.7% (1.5%-10.7%%)	9.6% (4.5%-17.2%)	10.9% (5.3-18.8%)	17.1% (9.0%-27.4%)	20.2% (10.6-31.9%)	20.2% (10.6-31.9%)
Anemia	0%	1.2% (0.1%-6.0%)	7.8% (3.2% -15.2%)	11.1% (5.1% -19.7%)	11.1% (5.1% -19.7%)	11.1% (5.1% -19.7%)

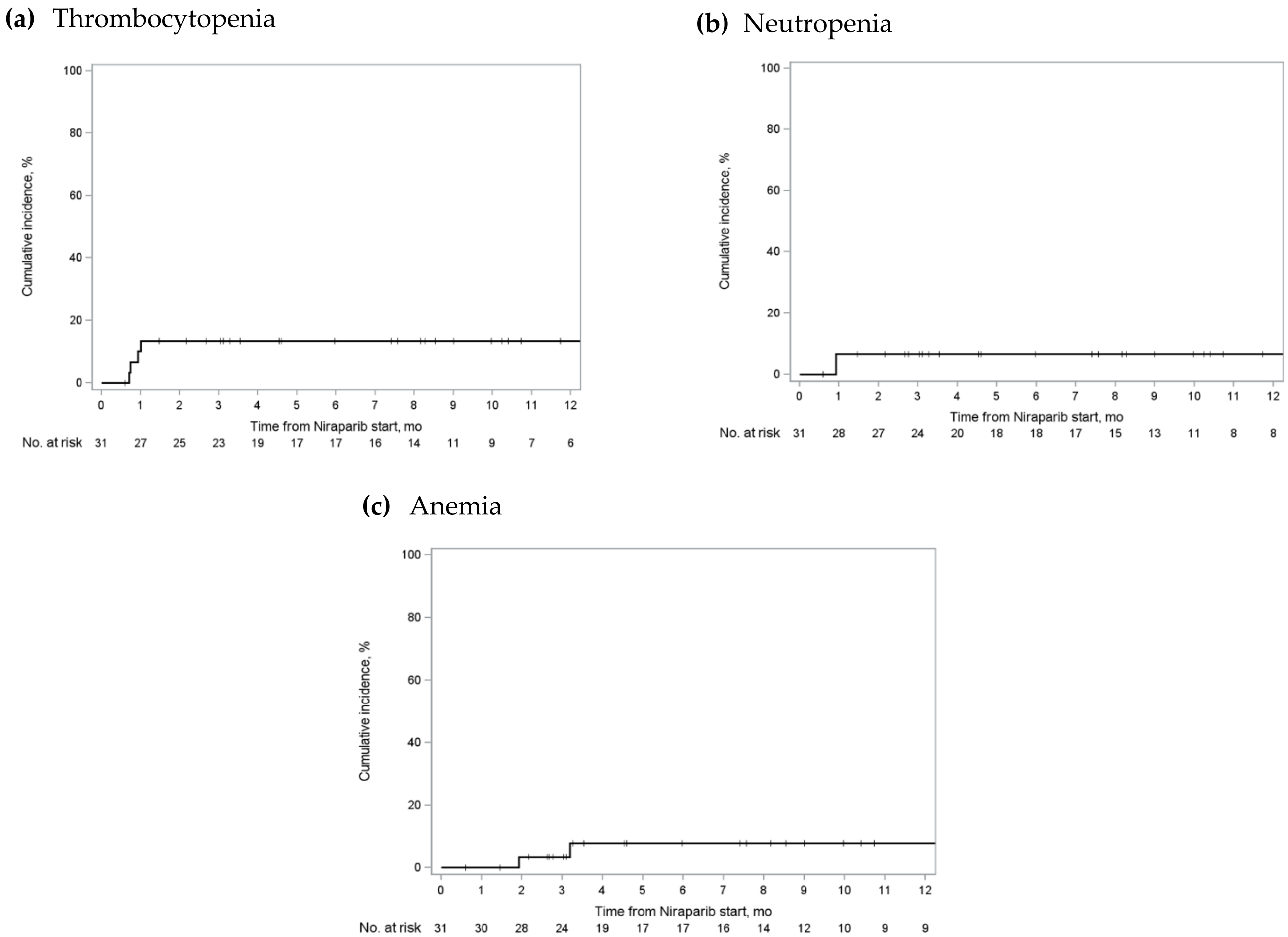

Figure S4. Cumulative incidence of grade 3/4 (**a**) thrombocytopenia; (**b**) neutropenia; and (**c**) anemia in Quebec.

Table S6. Cumulative incidence at various timepoints for grade 3/4 hematological adverse events in Quebec

Outcome	1 Month	2 Months	3 Months	6 Months	9 Months	12 Months
Thrombocytopenia	13.3% (4.1%-28.1%)	13.3% (4.1%-28.1%)	13.3% (4.1%-28.1%)	13.3% (4.1%-28.1%)	13.3% (4.1%-28.1%)	13.3% (4.1%-28.1%)
Neutropenia	6.7% (1.1%-19.4%)	6.7% (1.1%-19.4%)	6.7% (1.1%-19.4%)	6.7% (1.1%-19.4%)	6.7% (1.1%-19.4%)	6.7% (1.1%-19.4%)
Anemia	0%	3.4% (0.2%-15.2%)	3.4% (0.2%-15.2%)	7.8% (1.3%-22.6%)	7.8% (1.3%-22.6%)	7.8% (1.3%-22.6%)

(b) Thrombocytopenia

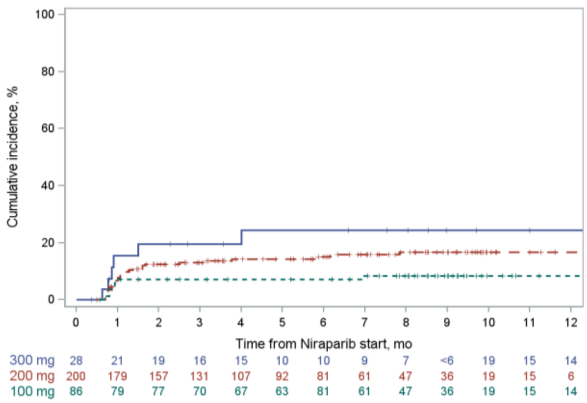

(a) Neutropenia

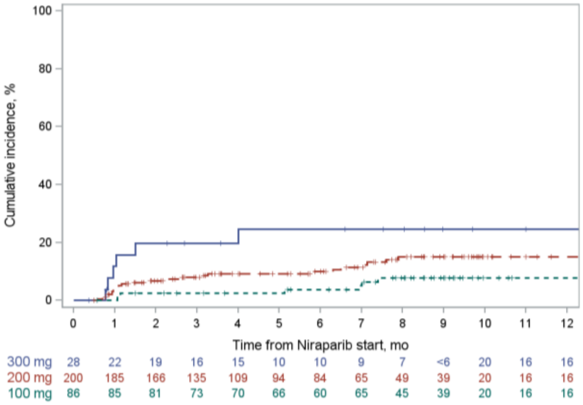

(c) Anemia

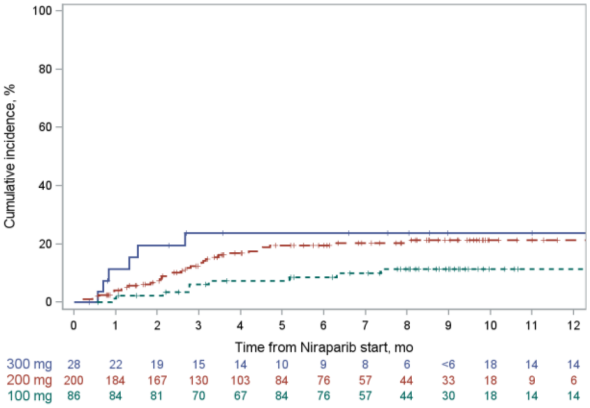

Figure S5. Cumulative incidence of grade 3/4 (**a**) thrombocytopenia; (**b**) neutropenia; and (**c**) anemia in Ontario, stratified by initial daily dose of niraparib. Note, the total number of patients included is 314, due to missing initial daily dose data and laboratory test data.

Table S7. Cumulative incidence at various timepoints for grade 3/4 hematological adverse events in Ontario, stratified by initial daily dose of niraparib.

Outcome	Initial Dose	1 Month	2 Months	3 Months	6 Months	9 Months	12 Months
Thrombocytopenia	100 mg/day	7.1% (2.9%-13.8%)	7.1% (2.9%-13.8%)	7.1% (2.9%-13.8%)	7.1% (2.9%-13.8%)	8.4% (3.7%-15.7%)	8.4% (3.7%-15.7%)
	200 mg/day	7.7% (4.5%-12%)	12.5% (8.3%-17.6%)	13% (8.7%-18.2%)	15% (10.3%-20.6%)	16.7% (11.6%-22.7%)	16.7% (11.6%-22.7%)
	300 mg/day	15.5% (4.7%-32%)	19.5% (6.9%-36.8%)	19.5% (6.9%-36.8%)	24.3% (9.5%-42.7%)	24.3% (9.5%-42.7%)	24.3% (9.5%-42.7%)
Neutropenia	100 mg/day	1.2% (0.1%-5.7%)	2.4% (0.4%-7.5%)	2.4% (0.4%-7.5%)	3.6% (1%-9.4%)	7.8% (3.2%-15.3%)	7.8% (3.2%-15.3%)
	200 mg/day	4.1% (1.9%-7.6%)	6.8% (3.8%-10.9%)	7.9% (4.6%-12.3%)	9.9% (6.1%-14.8%)	15% (9.8%-21.2%)	15% (9.8%-21.2%)
	300 mg/day	11.7% (2.8%-27.5%)	19.8% (7%-37.2%)	19.8% (7%-37.2%)	24.5% (9.6%-43.1%)	24.5% (9.6%-43.1%)	24.5% (9.6%-43.1%)
Anemia	100 mg/day	2.4% (0.4%-7.5%)	3.6% (0.9%-9.2%)	6% (2.2%-12.6%)	8.6% (3.7%-15.9%)	11.4% (5.5%-19.5%)	11.4% (5.5%-19.5%)
	200 mg/day	4.1% (1.9%-7.5%)	7.3% (4.2%-11.6%)	13.7% (9.1%-19.1%)	19.5% (13.9%-25.8%)	21.2% (15.3%-27.8%)	21.2% (15.3%-27.8%)

	300 mg/day	11.4% (2.8%-26.9%)	19.5% (6.9%-36.8%)	23.7% (9.3%-41.8%)	23.7% (9.3%-41.8%)	23.7% (9.3%-41.8%)	23.7% (9.3%-41.8%)
